# NF-YB family transcription factors in *Arabidopsis*: Structure, phylogeny, and expression analysis in biotic and abiotic stresses

**DOI:** 10.3389/fmicb.2022.1067427

**Published:** 2023-01-17

**Authors:** Bipasha Bhattacharjee, Vipin Hallan

**Affiliations:** ^1^Academy of Scientific and Innovative Research (AcSIR), Ghaziabad, India; ^2^Plant Virology Laboratory, Division of Biotechnology, CSIR-Institute of Himalayan Bioresource Technology (IHBT), Palampur, India

**Keywords:** *Arabidopsis thaliana*, *cis*-element, transcription factor, nuclear factor Y, abiotic and biotic stress

## Abstract

Nuclear factor-Y (NF-Y) transcription factors (TFs) are conserved heterotrimeric complexes present and widespread across eukaryotes. Three main subunits make up the structural and functional aspect of the NF-Y TFs: NF-YA, NF-YB and NF-YC, which bind to the conserved CCAAT- box of the promoter region of specific genes, while also interacting with each other, thereby forming myriad combinations. The NF-YBs are expressed differentially in various tissues and plant development stages, likely impacting many of the cellular processes constitutively and under stress conditions. In this study, ten members of NF-YB family from *Arabidopsis thaliana* were identified and expression profiles were mined from microarray data under different biotic and abiotic conditions, revealing key insights into the involvement of this class of proteins in the cellular and biological processes in *Arabidopsis*. Analysis of *cis*-acting regulatory elements (CAREs) indicated the presence of abiotic and biotic stress-related transcription factor binding sites (TFBs), shedding light on the multifaceted roles of these TFs. Microarray data analysis inferred distinct patterns of expression in various tissues under differing treatments such as drought, cold and heat stress as well as bacterial, fungal, and viral stress, indicating their likelihood of having an expansive range of regulatory functions under native and stressed conditions; while quantitative real-time PCR (qRT-PCR) based expression analysis revealed that these TFs get real-time-modulated in a stress dependent manner. This study, overall, provides an understanding of the AtNF-YB family of TFs in their regulation and participation in various morphogenetic and defense- related pathways and can provide insights for development of transgenic plants for trait dependent studies.

## 1. Introduction

Research on Nuclear Factor-Y (NF-Y) genes and their functional characterization have been reported decades back to the 1990s (Li et al., 1992; Albani and Robert, 1995). These multi-subunit TFs are found in plants, animals, and other eukaryotes like yeast. These are CCAAT box binding factors and comprise of three subunits NF-YA (CBF-B/HAP2), NF-YB (CBF-A/HAP3), and NF-YC (CBF-C/HAP5; Nardini et al., 2013) and are sometimes synonymously called Heme Activator Protein (HAP; Zhao et al., 2017). These TF subunits function by forming heterodimers, and further form diverse sub-families owing to its combinatorial intricacies (Petroni et al., 2012).

The structural units of the NF-YA, NF-YB and NF-YC are dissimilar. The NF-YA subunits are localised to nucleus and predominantly bind to the CCAAT-box present in the target gene promoters, with differential affinities (Calvenzani et al., 2012; Petroni et al., 2012). There are two α helix domains, i.e., A1 and A2, in which the A1 domain resides in the N-terminal core region and participates in the binding of the NF-YB and NF-YC subunits and consists of 20 amino acids. The C-terminal A2 domain comprises of specific-sequences for CCAAT binding and recognition (Petroni et al., 2012; Laloum et al., 2013; Nardini et al., 2013).

In contrast to the A subunit, the B and C subunits remarkably differ in structural composition, in that there is a Histone Fold Domain/Motif (HFD/HFM) which are formed by three α helices- α1, α2 and α3, and are imperative for protein-DNA and protein–protein interactions (Frontini et al., 2004; Kahle et al., 2005). The α1 helices in NF-YB contain presumed DNA-binding domains while in NF-YC the α1 helices comprise of conserved amino acids which may act as DNA binding domains (Laloum et al., 2013). To initiate the binding process and subsequent translocation to the nucleus, the NF-YBs must form heterodimers with NF-YC as all NF-YBs lack the nuclear localisation signal (NLS) in contrast to the NF-YA and NF-YC (Liu and Howell, 2010; Hackenberg et al., 2012). NF-YA, NF-YB and NF-Y-C are unable to form homodimer (indicated through yeast two hybrid assays) but have been known to form many heteromeric complexes (NF-YA-C or NF-YC-B) and differentially affect target genes and downstream mechanisms (Calvenzani et al., 2012; Hackenberg et al., 2012).

The NF-Y TFs have been shown to function and regulate downstream target genes *via* two distinct mechanisms: (1) NF-YB and NF-YC assemble in the cytoplasm as a heterodimer and together translocate to the nucleus where the complex binds to NF-YA and gets activated. This complex then binds to the CCAAT-box of target gene promoter to affect the gene expression (Hackenberg et al., 2012; Laloum et al., 2013). For example, the NF-YA4-NF-YB3-NF-YC2 complex binds to the promoter region of *BINDING PROTEIN 3* (*BiP3*) and activates the expression of endoplasmic reticulum (ER) stress genes in *Arabidopsis thaliana* (Liu and Howell, 2010), (2) Other TFs also form complex with NF-YB/YC and participates in regulation processes by binding to the *cis*-regulatory elements (CREs) in target gene promoters (Wenkel et al., 2006; Yamamoto et al., 2009; Kumimoto et al., 2010). For example, bZIP67 interacts with NF-YB9-NF-YC2 and directly bind to the ABA-response elements (ABRE) in the SUS2 and CRC (SUCROSE SYNTHASE 2 AND CRUCIFERIN C) promoter region to activate gene expression for seed development in *A. thaliana* (Yamamoto et al., 2009). NF-YAs are the chief binding subunits in most complex formation and gene expression. NF-Y complex, therefore, functions together with many factors to affect the regulation of different genes impacting a variety of regulatory pathways.

Biological functions of NF-Ys are diverse, with roles ranging from developmental pathways to environmental and pathogen stress. NF-YB9 or LEC1 (LEAFY COTYLEDON 1) was the first identified NF-Y subunit actively participating in embryogenesis and post-embryo development in *A. thaliana* (West et al., 1994; Lotan et al., 1998; Lee et al., 2003). In plants, NF-Y are involved in various biological processes, such as embryogenesis and endosperm formation (Stone et al., 2001; Kwong et al., 2003; Sun et al., 2014; Xu et al., 2016; Xiong et al., 2019; Niu et al., 2020), seed germination (Siriwardana et al., 2014; Liu et al., 2016; Sun et al., 2016), root morphogenesis (Ballif et al., 2011; Sorin et al., 2014; Zhou et al., 2020), flowering (Cao et al., 2014; Hou et al., 2014; Brambilla and Fornara, 2017; Lv et al., 2021), chloroplast biogenesis and photosynthesis (Miyoshi et al., 2003; Stephenson et al., 2010, 2011; Xuanyuan et al., 2017), starch biosynthesis (Bai et al., 2016), and fruit ripening (Li et al., 2016). NF-Ys have also been involved in abiotic and biotic stress response like drought stress (Nelson et al., 2007; Li et al., 2008; Lee et al., 2015; Palmeros-Suárez et al., 2015; Zhang et al., 2015; Wu et al., 2018; Sato et al., 2019; Zhou et al., 2020), temperature (Sato et al., 2014; Shi et al., 2014; Sato et al., 2016; Gyula et al., 2018; Sato et al., 2019) and salt stress (Chen et al., 2015; Feng et al., 2015; Ma et al., 2015), microbial stress (Combier et al., 2006, 2008; Hogekamp et al., 2011; Schaarschmidt et al., 2013; Battaglia et al., 2014; Laporte et al., 2014).

Transcriptional regulation is tightly controlled by the binding of TFs to CREs of target genes, thereby affecting metabolic or defence pathways (Kaur and Pati, 2016). These are regulatory motifs comprising of approximately 20 nucleotides present in the promoter regions. A variety of CREs are present, however in the case of NF-Ys, the predominant CRE is the *CCAAT* box. A variety of CREs and enhancer/repressor elements are present in these regions like TATA box, GC region, which are essential for expression/repression of gene expression (Wittkopp and Kalay, 2012). Computational methods of promoter characterization are a popular and cost-effective alternative to the current wet lab techniques and can give very good initial datasets for gene promoter studies and functional characterization. Some common web based analytical tools that are widely used for identification of CREs in promoters include PLACE (Higo et al., 1999), PlantCARE (Lescot et al., 2002), AGRIS (Davuluri et al., 2003), TRANSFAC (Matys et al., 2003) and PlantPAN (Chow et al., 2016). This study discusses structure, phylogeny, expression analysis of NF-YB family of TFs in *Arabidopsis*. Additionally, we identified potential CREs primarily related to stress conditions in NF-YB gene promoters. Collectively, our findings provide a plethora of information on potential roles of NF-YBs in stress biology, which could be deeply investigated to arm the plants against harsh responses of the environment.

## 2. Materials and methods

### 2.1. Data retrieval

AtNF-YB (B1–B10) subunits (10 in total) were identified, and data was extracted from the *Arabidopsis* information resource (TAIR).^1^ NCBI was used for sequence verification and nucleotide BLAST analysis was performed to verify the sequences. Coding sequences (CDS) were identified, and their corresponding mRNA and protein sequences were obtained.

### 2.2. Identification and characterization of AtNF-YB genes

Physicochemical characteristics of *A. thaliana* NF-YB proteins such as their molecular size, isoelectric point (pI), instability index, aliphatic index, and grand average of hydropathicity (GRAVY) were calculated by ProtParam (Gasteiger et al., 2005).^2^ Putative subcellular localisation was predicted using WoLF PSORT (Horton et al., 2007)^3^ and CELLO v.2.5: subCELlular Localization predictor (Yu et al., 2006)^4^ and PlantPLoc (Chou, 2005; Chou and Shen, 2006; Chou and Shen, 2007, 2008, 2010). A total of 10 NF-YB subunits have been reported in *A. thaliana*. Chromosome maps of the genes were constructed by “chromosome map” tools in TAIR database.^5^ The server of gene structure display has been used to visualise intron/exon organisation of genes (Hu et al., 2015).^6^

### 2.3. Sequence alignment, generation of phylogenetic tree and clusters

Hidden Markov model (HMM) profile was made to confirm the existence of domains conserved within the AtNF-YB subunits using the tool InterProScan.^7^ RegsiteDB was used to identify regulatory elements present in the TFs. Multalin programme was used for multiple sequence alignment using the default parameters^8^ and phylogenetic tree was constructed using bootstrap values at 1000 iterations using MEGA X software^9^ and NGPhylogeny (Dereeper et al., 2008).^10^ Calculations done based on neighbour-joining (NJ) method (Kumar et al., 2016, 2018). UPGMA (unweighted pair group method with arithmetic mean) was used to trace out the genetic distance and forming evolutionary history-based clusters (Sneath and Sokal, 1973). The evolutionary distances, measured in the number of amino acid substitutions per site, were calculated using the Poisson correction technique (Zuckerkandl and Pauling, 1965). The MEME programme which provides motif discovery using algorithms which are both probabilistic and discrete (MEME and STREME) and MOTIF Search were utilised for the identification of conserved structures of the AtNF-YB proteins (Bailey and Elkan, 1994). The *p*-value threshold was kept at <0.05.

### 2.4. Analysis of CREs

AtcisDB, which is an *Arabidopsis* resource bank for gene regulatory information, was used for extracting promoter sequences of individual NF-YB TFs. PlantCare (Lescot et al., 2002)^11^ and AGRIS (Davuluri et al., 2003)^12^ were used for identification and investigation of the CAREs. Locus ID, gene model, and gene ontology from MIPS, TIGR, TAIR, and SALK was detected. The promoter region evaluated comprised of 5′UTR (untranslated region) along with core and distal promoter regions. TFBs (transcription factor binding sites) involved in various biotic and abiotic stress responses, were identified, and studied.

### 2.5. Expression analysis AtNF-YB genes

AtNF-YB gene expression data was inferred by Genevestigator (Nebion AG, Zurich, Switzerland)^13^ from its *Arabidopsis thaliana* database. Differential gene expression was assessed using the development and perturbations tools under normal conditions as well as biotic and abiotic stresses, respectively. Down/upregulation of genes was represented as fold change ratio and 2.0-fold was kept as a benchmark for fold difference in gene expression values. For the evaluation, AT_AFFY_ATH1-0 microarray data was used as reference transcriptome compendium with col-0 as reference genotype. Red/Green colour scheme was used to represent the heatmap, where red showed down-regulation and green showed up-regulation of the genes. All 10 AtNF-YBs were scrutinised for selected abiotic and biotic stressors under certain experimental conditions and heat maps were generated as a comparative transcript fold change response between all the subunits (Zimmermann et al., 2004). Data inferred from Genevestigator were filtered using a 2-fold change cutoff and *p* < 0.05.

### 2.6. Plant materials and growth conditions

*Arabidopsis thaliana* ecotype Columbia (col-0) was used for all the qRT-PCR experiments for functionally validating expression of AtNF-YB family of genes in control as well as treated conditions. *Arabidopsis thaliana* seeds were surface sterilised for 15 min in 70% ethanol and 5% (w/v) bleach and subsequently washed with water for about six times. Seeds were then germinated in half-strength Murashige and Skoog (MS) media in a growth chamber at 23°C and 16 h/8 h light and dark cycle, respectively. Two-week-old seedlings were used for abiotic stress treatments. For heat and cold stress conditions, seedlings were subjected to 38°C and 4°C for 1.5 h and flash frozen for RNA isolation. To analyse the expression of the TFs, salt stress (NaCl 50 mM and 150 mM), osmotic stress (Mannitol 50 mM), phytohormone stress (IAA 1 μM, JA 10 μM, SA 10 μM, ABA 10 μM and MeJA 1 μM) were given exogenously for a duration ranging from 1 to 3 h. RNA was isolated immediately after stress treatments; untreated seedlings were used as control.

### 2.7. RNA extraction and expression analysis

Total RNA was isolated from control and treated samples using TRIzol reagent (Invitrogen, Carlsbad, CA, United States). A Nanodrop2000 instrument (New England Biolabs, MA, United States) was used to measure RNA concentration and purity estimation following the manufacturer’s instructions, along with gel electrophoresis. Verso cDNA Synthesis Kit (Thermo Fisher Scientific Inc., Waltham, MA, United States) was used for the reverse transcription of 1 μg RNA per sample according to kit protocol. Quantitative Real Time PCR was performed using the Himedia Insta Q96 real time PCR machine (Mumbai, Maharashtra, India) and carried out with the final volume of 10 μl in triplicates using the cycling conditions: initial denaturation at 95°C for 3 min, followed by 40 cycles of 95°C at 15 s and 60°C for 1 min and finally a melt curve analysis. Fold changes were computed using the 2^−ΔΔCt^ method (Livak and Schmittgen, 2001) and the Δ*C*t values were normalised to an internal control At18s rRNA. Each sample was analyzed in triplicate. The primers (Supplementary File 3) were custom-designed and procured from Integrated DNA Technologies (IA, United States). Each reaction comprised of 5 μl DyNAmo ColorFlash SYBR Green qPCR mix (Thermo Fisher Scientific Inc., Waltham, MA, United States), 3 μl of cDNA (1:10 dilution) and 0.3 μM of forward and reverse primers each. The fold changes of treated plants relative to the control were used to express all values.

### 2.8. Data analyses

Significant differences between experimental and control groups under different stress conditions were assessed using one-way ANOVA followed by Dunnett’s post-hoc test for multiple comparisons. All analyses were performed using GraphPad Prism 9.4.1 (GraphPad Software Inc., CA, United States). Data are expressed as the mean group value ± standard error mean (SEM) from at least three independent experiments. *p* < 0.05 was considered statistically significant where *p < 0.05; **p < 0.01; ***p < 0.001; ****p < 0.0001.

## 3. Results

### 3.1. Structural analysis and characterization of AtNF-YB genes

**Figure 1:**
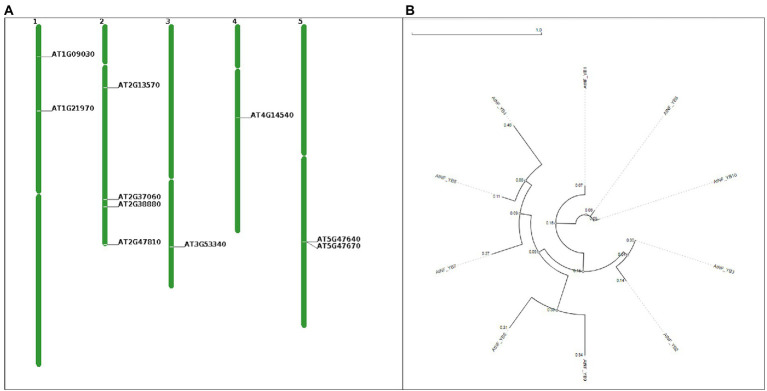
**(A)** Chromosome map tools generated visual depiction indicating positions of AtNF-YB1 to AtNF-YB10, in the *Arabidopsis thaliana* chromosomes. Locus tags in the image are represented as AT2G38880 (AtNF-YB1), AT5G47640 (AtNF-YB2), AT4G14540 (AtNF-YB3), AT1G09030 (AtNF-YB4), AT2G47810 (AtNF-YB5), AT5G47670 (AtNF-YB6), AT2G13570 (AtNF-YB7), AT2G37060 (AtNF-YB8), AT1G21970 (AtNF-YB9) and AT3G53340 (AtNF-YB10). **(B)** Phylogenetic circular tree of AtNF-YB family members constructed using NGPhylogeny.fr and visualised using Phylogenetic tReE viSualisaTiOn (PRESTO) using the maximum likelihood method, using the FastME workflow in automatic default parameters. All 10 protein sequences were extracted from NCBI.

AtNF-YB subunits were retrieved from TAIR and characterised as represented in Table 1. Length of the coding sequence (CDS) vary from 420 bp (NF-YB4) to 717 bp (NF-YB9). Chromosomal location of 10 NF-YBs, CDS and protein are presented in Figure 1A and Table 1. Chromosome 2 has four NF-YB genes-NF-YB1/B5/B7/B8 present at locus AT2G38880, AT2G47810, AT2G13570, and AT2G37060, respectively. Further, chromosomes 1 and 5 have two (NF-YB4/B9 and NF-YB2/B7 at positions AT1G09030/AT1G21970 and AT5G47640/AT2G13570) each, followed by one NF-YB gene each in chromosomes 3 and 4 (NF-YB10 at AT3G53340 and NF-YB3 at AT4G14540, respectively), according to the chromosome map tool in TAIR (Figure 1A).

**Table 1:** Characteristics of *Arabidopsis thaliana* NF-YB genes: chromosome location, locus ID and basic sequence characteristics.

Transcription factor name	Chromosome location	Locus ID	Gene CDS length (bp)	Amino acid length (aa)
AtNF-YB1	2	AT2G38880	495	164
AtNF-YB2	5	AT5G47640	573	190
AtNF-YB3	4	AT4G14540	486	161
AtNF-YB4	1	AT1G09030	420	139
AtNF-YB5	2	AT2G47810	483	160
AtNF-YB6	5	AT5G47670	705	234
AtNF-YB7	2	AT2G13570	648	215
AtNF-YB8	2	AT2G37060	522	173
AtNF-YB9	1	AT1G21970	717	238
AtNF-YB10	3	AT3G53340	531	176

Physicochemical characteristics such as pI, GRAVY, instability index, aliphatic index, and subcellular localization are presented in Table 2. As results indicate, corresponding putative precursor proteins of AtNF-YBs varied from 15.74 kDa (AtNF-YB4) to 26.1 kDa (AtNF-YB6) with their corresponding pI values at 6.91 to 6.66. Subcellular localization analysis placed all the 10 predicted AtNF-YB subunit proteins exclusively within the nucleus.

**Table 2:** Basic properties of AtNF-YB1-B10: physicochemical attributes calculated using the ProtParam tool.

Gene name	Protein Mol.Wt. (kDa)	pI	Aliphatic index	Instability index	Stability	GRAVY	Localization	Localization steps
AtNF-YB1	18	4.89	70.79	36.36	Stable	−0.556	Nucleus	Intracellular → Nucleus or Cytoplasm → Nucleus
AtNF-YB2	20.5	6.0	49.32	37.36	Stable	−0.905	Nucleus	Intracellular → Nucleus or Cytoplasm → Nucleus
AtNF-YB3	17.18	5.81	51.55	33.26	Stable	−0.720	Nucleus	Intracellular → Nucleus or Cytoplasm → Nucleus
AtNF-YB4	15.74	6.91	63.17	39.58	Stable	−0.963	Nucleus	Intracellular → Nucleus or Cytoplasm → Nucleus
AtNF-YB5	18.1	6.44	60.31	37.85	Stable	−0.870	Nucleus	Intracellular → Nucleus or Cytoplasm → Nucleus
AtNF-YB6	26.1	6.66	58.76	47.15	Unstable	−0.643	Nucleus	Intracellular → Nucleus or Cytoplasm → Nucleus
AtNF-YB7	24.6	5.98	53.02	65.47	Unstable	−1.147	Nucleus	Intracellular → Nucleus or Cytoplasm → Nucleus
AtNF-YB8	18.99	6.43	53.64	48.60	Unstable	−0.821	Nucleus	Intracellular → Nucleus or Cytoplasm → Nucleus
AtNF-YB9	26.0	5.68	61.85	44.79	Unstable	−0.638	Nucleus	Intracellular → Nucleus or Cytoplasm → Nucleus
AtNF-YB10	19.15	5.54	51.53	49.06	Unstable	−0.807	Nucleus	Intracellular → Nucleus or Cytoplasm → Nucleus

### 3.2. Phylogenetic analysis and classification of AtNF-YB genes

To understand the characteristics of the AtNF-YB class of proteins, phylogenetic analysis was performed using a sequence-based approach. MEGA-X, assisted by NGPhylogeny tool was employed for the analysis and calculations while UPGMA method assisted in genetic distance measurement using default parameters (Figure 1B). The branch lengths on the tree which are shown to scale are in the same units as the evolutionary distances used to estimate the phylogenetic tree; 10 amino acid sequences were examined in this investigation. For each sequence pair, all unclear places were eliminated (pairwise deletion option). Variations were observed in the phylogram evolutionary analyses, performed utilising MEGA11 (Tamura et al., 2021). A circle tree was constructed to understand evolutionary similarity and divergence. Similar external nodes of AtNF-YBs are as follows: B2 and B3, B9 and B6, B4, B5 and B7 sharing a common ancestor, and B8 and B10 sharing a common internal node, with similarity with B1. The distances along with the rooted circular phylogenetic tree are represented in Figure 1B.

**Figure 2:**
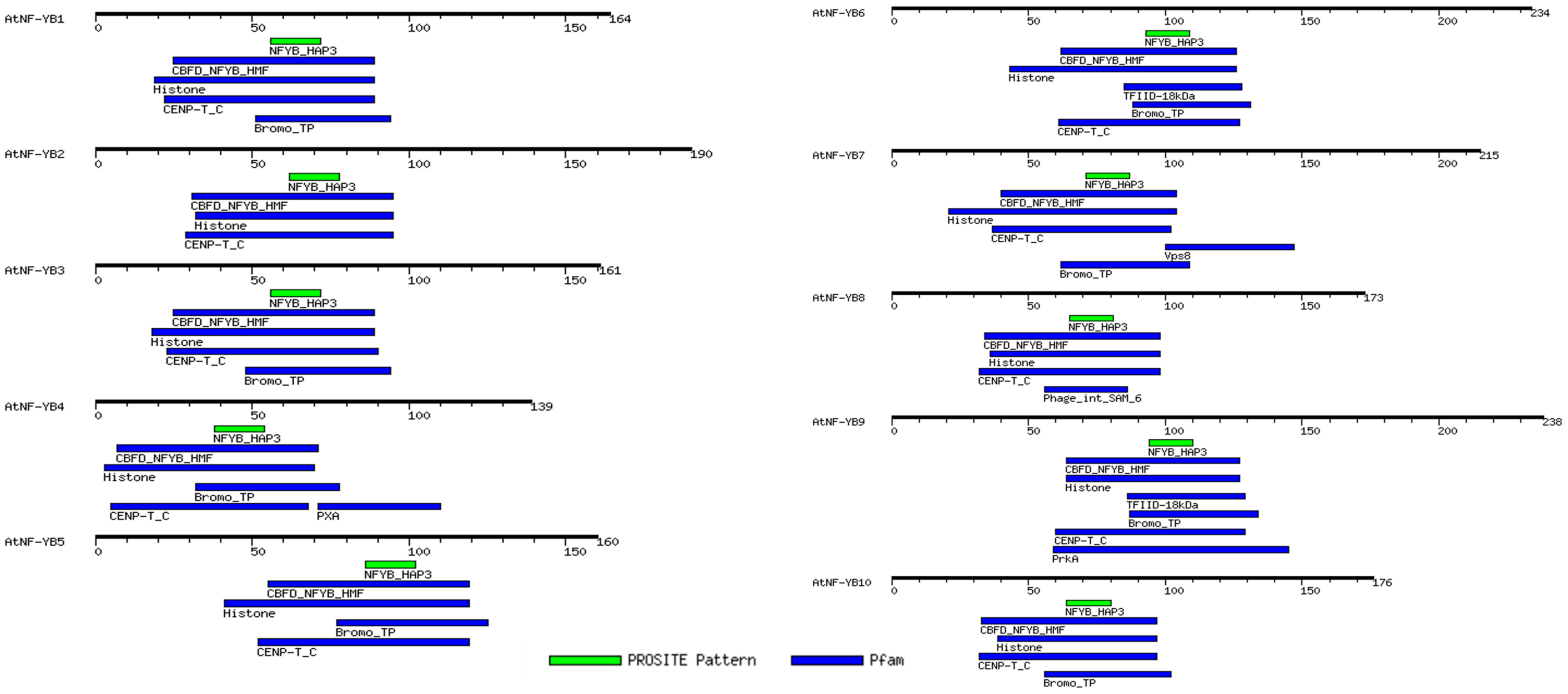
MotifFinder tool, used for identification of motifs on each AtNF-YB gene. Colour coded motif symbol represents the motifs of the highest density and prevalence. Default parameters of E-value at 1.0, and utilisation of Pfam and PROSITE databases were used and used for analysis and visualisation.

Similar motifs and types were detected, using the MEME programme and MOTIF search tool, in all the genes of this family and their positions are highlighted (Figure 2). Most common motifs comprise of histone-like TF (CBF/NF-Y) domain, which are primarily a family of archaebacterial histones and histone-like TFs present in eukaryotes, CENP-T_C domain, which is a chromatin binding vertebral kinetochore protein family, Bromo_TP domain which is found in eukaryotes and has a histone like fold which putatively binds DNA and histone binding domain which essentially binds to DNA, leading to gene regulation. Some other domains unique to a few members are the PXA domain in NF-YB4, which is probably required for filament formation/protein–protein interactions; TFIID-18 kDa motif harbouring within NF-YB6, which along with TATA-binding domain (TBP) mediates transcription of DNA to RNA; Vsp8 binding domain within NF-YB7 which is a site of recognition for vacuolar protein sorting-associated protein 8 homologue which helps enable endosomal vesicle fusion *via* metal ion binding activity; Phage integrase SAM like domain in NF-YB8 which are conserved for an enzyme group that are integral in catalysis of DNA breakage and re-joining; and a PrkA domain within NF-YB9 which is specific for bacterial and archaeal serine kinases. Most of the motifs present are generally those which are associated with transcriptional regulation of genes (Table 3).

**Table 3:** Major motifs present in the 10 AtNF-YB proteins and their corresponding positions in the individual proteins, represented using the MOTIF Search tool.

Protein name	Name of major motifs	Position
AtNF-YB1	CBFD_NFYB_HMF	25..89
	Histone	19..89
	CENP-T_C	22..89
	Bromo_TP	51..94
AtNF-YB2	CBFD_NFYB_HMF	31..95
	Histone	32..95
	CENP-T_C	29..95
AtNF-YB3	CBFD_NFYB_HMF	25..89
	Histone	18..89
	CENP-T_C	23..90
	Bromo_TP	48..94
AtNF-YB4	CBFD_NFYB_HMF	7..71
	Histone	3..70
	Bromo_TP	32..78
	CENP-T_C	5..68
	PXA	71..110
AtNF-YB5	CBFD_NFYB_HMF	55..119
	Histone	41..119
	Bromo_TP	77..125
	CENP-T_C	52..119
AtNF-YB6	CBFD_NFYB_HMF	62..126
	Histone	43..126
	TFIID-18 kDa	85..128
	Bromo_TP	88..131
	CENP-T_C	61..127
AtNF-YB7	CBFD_NFYB_HMF	40..104
	Histone	21..104
	CENP-T_C	37..102
	Vps8	100..147
	Bromo_TP	62..109
AtNF-YB8	CBFD_NFYB_HMF	34..98
	Histone	36..98
	CENP-T_C	32..98
	Phage_int_SAM_6	56..86
AtNF-YB9	CBFD_NFYB_HMF	64..127
	Histone	64..127
	TFIID-18 kDa	86..129
	Bromo_TP	87..134
	CENP-T_C	60..129
	PrkA	59..145
AtNF-YB10	CBFD_NFYB_HMF	33..97
	Histone	39..97
	CENP-T_C	32..97
	Bromo_TP	56..102

Databases like Pfam and PROSITE format were used and cut off E-value was 1.0, as per default settings.

### 3.3. *In silico* retrieval and analysis of *CREs* in AtNF-YB genes

CREs are essential part of promoters which are vital for binding of TFs, which then control regulation of target genes (Kaur et al., 2017). Using the locus ID, all 10 NF-YBs were subjected to AGRIS (*Arabidopsis* gene regulatory information server) and AtcisDB for data generation of the promoter regions of each gene, sourced from TAIR. Promoter information, position within chromosomes, promoter type, and source are presented in Table 4. The predicted range of the promoters using AtcisDB (Palaniswamy et al., 2006) were 300 bp-1.5kbp.

**Table 4:** Description of promoters and their chromosomal location utilising atcisDB from AGRIS database.

Promoter ID	Description	Chromosomal location	Promoter type	Source
AtNF-YB1	CCAAT-binding transcription factor subunit	Chr2:16242551–16245553	Predicted	TAIR
AtNF-YB2	CCAAT-binding transcription factor subunit	Chr5:19325073–19326452	Predicted	TAIR
AtNF-YB3	CCAAT-binding transcription factor subunit A (CBF-A)	Chr4:8343877–8344611	Predicted	TAIR
AtNF-YB4	CCAAT-binding transcription factor subunit	Chr1:2909032–2909454	Predicted	TAIR
AtNF-YB5	CCAAT-box binding transcription factor	Chr2:19590686–19593490	Predicted	TAIR
AtNF-YB6	CCAAT-HAP3	Chr5: 18712512–18713512	Predicted	TAIR
AtNF-YB7	CCAAT-box binding transcription factor	Chr2:5663570–5664220	Predicted	TAIR
AtNF-YB8	CCAAT-box binding transcription factor	Chr2:15580554–15583136	Predicted	TAIR
AtNF-YB9	CCAAT-box binding factor HAP3 homologue	Chr1:7729605–7731645	Predicted	TAIR
AtNF-YB10	Transcription factor NF-Y CCAAT-binding – like protein	Chr3:19786968–19788580	Predicted	TAIR

Several different types of CREs were identified, including: auxin responsive (AuxRE-core), wound responsive motif (WUN motif), gibberellin-responsive (GARE-motif, TATC-box), MeJA-responsive (TGACG-motif, CGTCA-motif), low-temperature receptive (LTR), oxidative stress responsiveness (as-1), abscisic acid-responsive (ABRE), stress sensitivity (TC-rich repeats, G-box, W-box), drought responsive MYB (MBS), ethylene-responsive (ERE), salicylic acid responsiveness (TCA-element), and anaerobic responsive element (ARE), amongst others.

#### 3.3.1. *Cis* elements: Stress related

**Figure 3:**
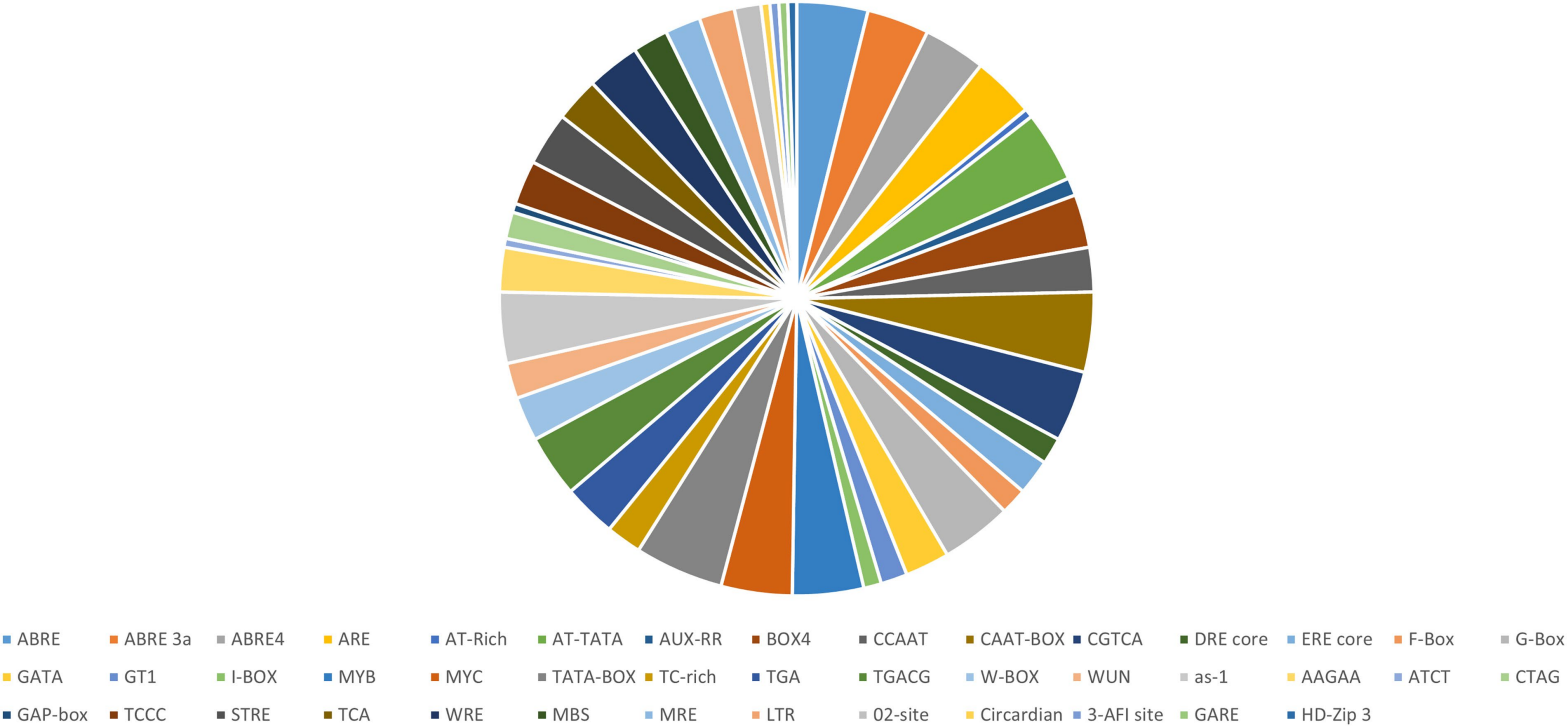
Pie distribution of AtNF-YB *cis-*elements identified using PLANTCARE, represented according to the percentage of prevalence of major *cis-*elements in the promoter regions of the AtNF-YB family of TFs.

Abiotic stress tolerance is a complex issue since it can occur simultaneously and has an impact on growth at various developmental stages of a plants (Park et al., 2003). Different elements related to several stress responses like light, heat, temperature, oxidation, drought, and wound were identified in each NF-YB gene of *A. thaliana* using the PLANTCARE server (Lee et al., 2015; Figure 3). We found that the promoters of AtNF-YB1/5/8/9/10, contained CREs related to defence response, W-box like repeat elements; and MBS, which is related to drought responsiveness, was abundant in AtNF-YB5/6/9/10.WUN motif, which is a wound responsive element essential in plant stress tolerance, is present in AtNF-YB1/4/7/10. AREs, which are evolutionary significant and play an essential role in diverse functions including primary and secondary metabolism, signalling and stress response are copiously present in AtNF-YB1, AtNF-YB3, AtNF-YB5, AtNF-YB7, AtNF-YB8, AtNF-YB9 and AtNF-YB10.

**Figure 4:**
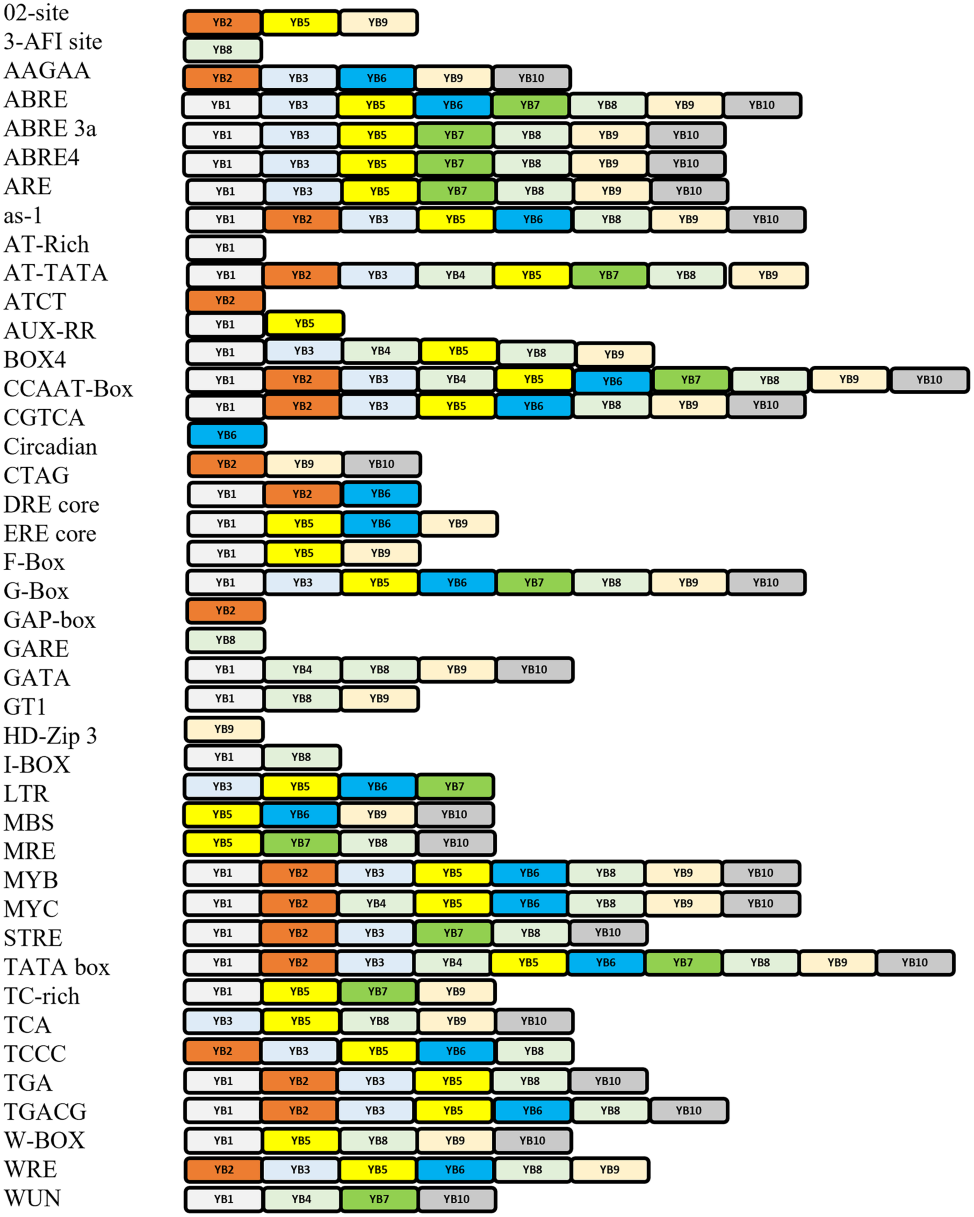
Representation of *cis*-elements typically occurring in the promoters of the AtNF-YB family of TFs. *Cis*-regulatory elements (CREs) are on the left along with corresponding colour coded AtNF-YB subunits.

Amongst abiotic stress responses, the as-1 (activation sequence-1), which is a salicylic acid (SA), and auxin-responsive element (AuxRE), are present across AtNF-YB1, AtNF-YB2, AtNF-YB3, AtNF-YB5, AtNF-YB6, AtNF-YB8, AtNF-YB9 and AtNF-YB10. ABREs are widespread in almost all the AtNF-YBs, except AtNF-YB3/B4/B6. G-box element is an important motif, involved in light, ABA, methyl jasmonate (MeJA), and ethylene responses, is also abundantly present in AtNF-YB1/B3/B5/B6/B7/B8/B9/B10. A WRKY family-specific CRE, called the W-box, has essential roles in abiotic/biotic stress and senescence, and is present abundantly in the promoters of AtNF-YB1/B5/B8/B9/10. Other oxidative stress elements include ERE core, present in AtNF-YB1/B5/B6/B9/B10; and AREs which are anaerobic responsive elements functional in induction of anaerobic response and contain GC/GT motifs are spanned across AtNF-YB1/B3/B5/B7/B8/B9/B10. Interestingly, GT-motifs, which are commonly found in the promoter regions of anaerobically induced plant genes, is putatively understood to have functions in drought and lower oxygen levels. Another motif, TGACG is a MeJA responsive element which is well characterised and spread acutely on almost all the AtNF-YBs (Figure 4). *Cis*-elements correlated with light responsiveness and stress like Box4, Gap box, ATCT motif, CATT motif and GATA are associated with all NF-YB promoters of *A. thaliana* (Figure 4). Calcium being an important small molecule regulator of biological functions in plants is an indispensable component of defence mechanisms during pathogen attack on plants. When pathogen attack activates the defence responses of plants, a major event that takes place is the SA accumulation in cells which potentially affects its redox potential (Garretón et al., 2002). As these events are controlled by the conversion of the inactive NPR1 to active NPR1, the activated monomers bind to the TGA *cis* regions in the nucleus, prompting the binding between the SA responsive elements of the pathogenesis related (PR) genes and TGA motifs, successfully launching systemic acquired resistance (SAR; Fan and Dong, 2002). TGA motifs are conserved in the subunits AtNF-YB1/B2/B3/B5/B8/B10, indicating their potential roles in biotic pathogenesis. Promoter motifs regulated by calcium, present in the AtNF-YB family include those of ABRE (abscisic acid responsive element), W-box (fungal, ooomycte and bacterial elicitor) and DRE (drought responsive). Although all classes of ABREs are widely present, DRE and W-box elements are sparsely scattered within the AtNF-YB family. W-box elements, conserved in the subunits AtNF-YB1/B5/B8/B9/B10, are also major elements in PR1 and NONEXPRESSER OF PR GENES1 (NPR1) gene expression regulation, therefore are key players in biotic stress response. Another biotic stress response element, the MRE-like, which is a site for fungal elicitor induced protein-DNA interactions and are present in the promoters of AtNF-YB5/B7/B8/B10 (Lois et al., 1989). These are MYB recognition elements that are diverse in functionality and participate in a variety of plant defence responses. Since hormones such as SA and JA are amongst the most recognised signal molecules participating in transcriptional mobilisation of PR proteins, many CREs participating in hormonal signalling may putatively participate in plant pathogen response mechanisms. CREs involved in light signalling and its consequent participation in biotic stress responses are also critical in understanding the participation of these elements in triggering variable stress responses in plants against pathogen attack.

#### 3.3.2. *Cis* elements: Growth and development related

Growth responsive CREs like AuxRE-core, GARE-motif, TATC-box, CAT box, F-box, O2-site, circadian, MeJA-responsive (TGACG-motif, CGTCA-motif) are also present across almost all NF-YB family, as tabulated in Figure 4. O2-site, an important *cis-*element which has a known functional role in zinc metabolism is present only in AtNF-YB2/B5/B9. *Cis*-elements related to circadian rhythm are only present in AtNF-YB6. The GARE box, which is a GA-regulatory element for hydrolase genes in germinating seeds is present only in AtNF-YB8, so is the steroid receptor 3-AF1 site. The HD-Zip3 receptor, similarly, is present in AtNF-YB9 only and it functions to support secondary cell wall formation in plants, leading to growth and development. Compared to the *cis*-elements responsive to stress, those of development are more sparsely located in the promoters of the AtNF-YB genes.

### 3.4. Expression profiles of AtNF-YBs in different developmental stages

**Figure 5:**
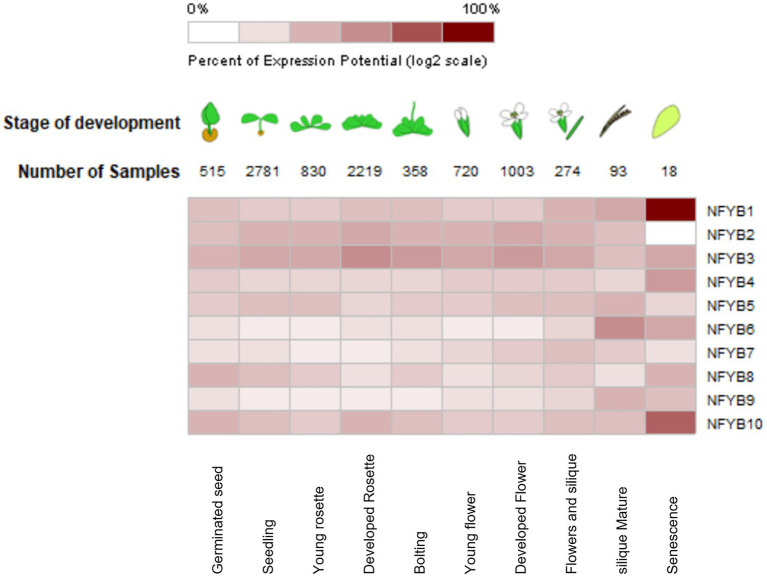
Heat-map showing the relative expression levels of AtNF-YB genes at different developmental stages of *Arabidopsis thaliana* compiled from the microarray datasets. Values and colour coding was based on the expression levels, where dark colouring constitutes highest expression as indicated by the scale and is the result of clustering by similarity of expression.

Owing to the availability of thousands of microarray datasets in open source, meta-analysis was conducted through Genevestigator on *A. thaliana* for all the NF-YB genes. Changes in gene expression levels under abiotic and biotic stressors and various developmental stages were examined, which indicate the involvement of AtNF-YB family in various biological processes. Senescence is a complex biological process, which essentially is the time when plants are towards the end of their lifespan and involves genes which participate in mobilisation of soluble minerals and cell degradative functions. It was found that AtNF-YB1 showed the strongest expression level at the stage of senescence followed by AtNF-YB10. AtNF-YB3/B4/B6 showed consistent strong expression during the senescence stage of *A. thaliana* development, inferring upon the fact that this family of genes has a presumed role in senescence regulation. AtNF-YB1/10 has a putative function in regulation of genes during cell death and senescence, based on the microarray data. AtNF-YB2/B5/B8/B10 have same level of expression in the seedling stage and AtNF-YB3/B8/B10 displayed high expression levels in germinating seedlings, possibly participating in embryogenesis and seed development (inferred from the AT_AFFY_ATH1-0 dataset). AtNF-YB3 showed strong expression levels in the developed rosette stage, bolting, and flowering stages, suggesting its role to the flowering pathways. A multi-pathway role was detected with consistently higher expression in AtNF-YB2 from seed up to matured siliques. These results suggest that NF-YB family participates in a diverse functional role in plant development, from seed germination to senescence (Figure 5).

### 3.5. Expression profiles of AtNF-YBs in response to phytohormones, abiotic and biotic stresses

TFs have a multidimensional role in stress response and most of NF-YBs across different plant species have been severely underrepresented when it comes to stress biology studies. Expression of all 10 AtNF-YBs were scrutinised for selected phytohormones, abiotic and biotic stressors using the open access data available for col-0 ecotype of *A. thaliana* and heat maps were generated. AT_AFFY_ATH1-0 microarray data was used for this analysis.

Abiotic stress is one of the most common kind of stressors, widespread environmentally, dynamically regulating plant physiology in day-to-day life. Common abiotic elements include cold, heat, drought, osmotic stress, UV radiation, and waterlogging, amongst others. These pose severe threats to agriculture as they are a huge part of crop yield loss, worldwide. Selected experimental setups were extracted from the available genome array data and expression patterns of AtNF-YBs were analysed for their and putative roles in various stress responses.

**Figure 6:**
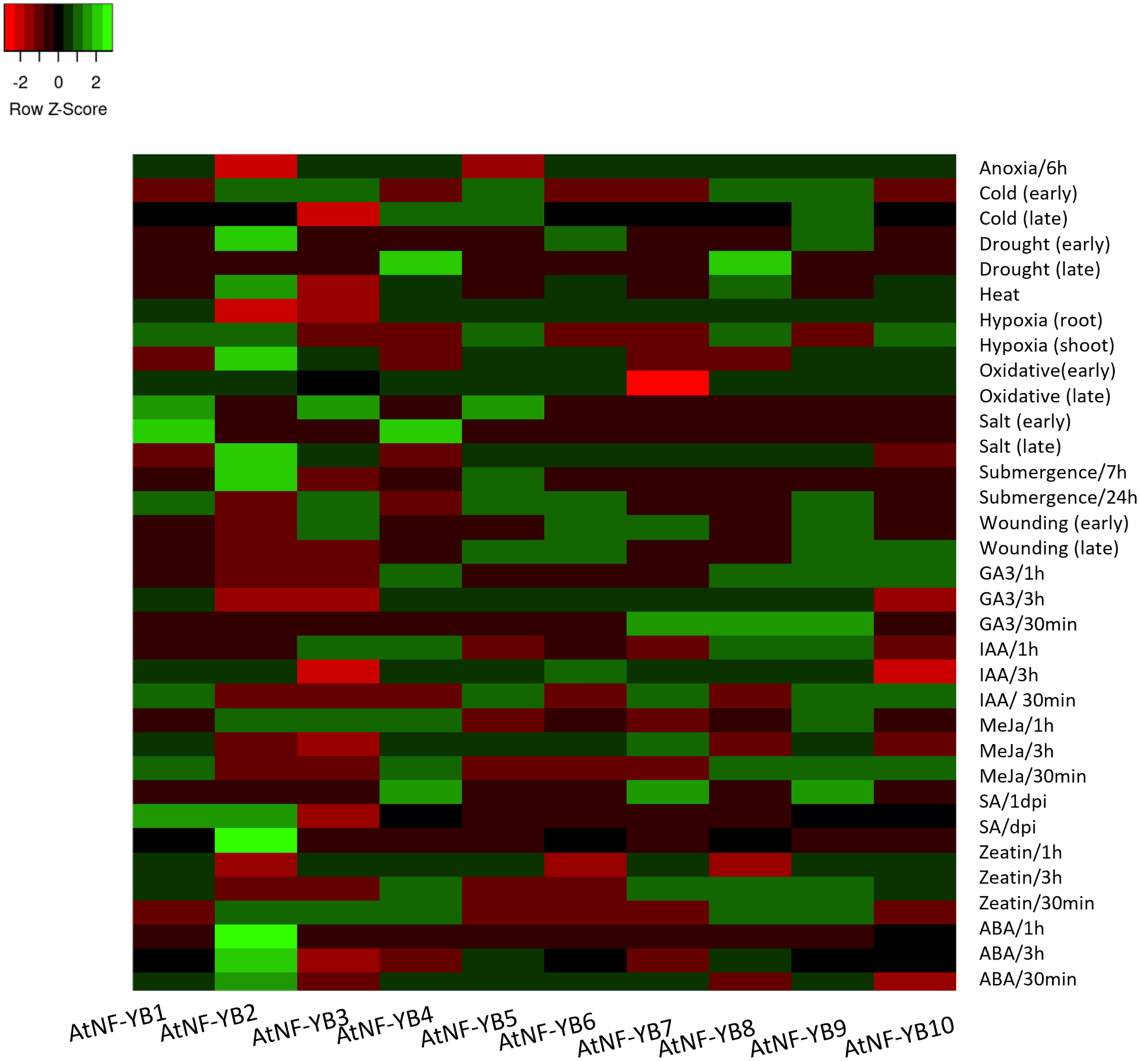
Heat map showing the expression profiles of 10 AtNF-YB genes under different abiotic stress conditions. The colour scale is representative of the log2- transformed relative gene expression that is observed under control conditions. Red to green colours denote low to high relative expression levels.

Being obligate aerobes, hypoxia, and anoxia (oxygen deficiency and absence, respectively) can cause a histrionic reprogramming of the plant molecular pathways and cause ecological stress. Dark and low oxygen conditions in *Arabidopsis* seedlings have been shown to downregulate AtNF-YB2 and AtNF-YB5, as opposed to the heightened expression of the rest of the family, which may infer upon that this family of TFs may be involved in positive regulation in case of low oxygen contents in the environment (Figure 6). Cold stress is another abiotic factor, which elicits a TF response, which in turn binds to cold stress responsive genes that help in the cold coping mechanisms. *Arabidopsis thaliana* seedlings (16-day-old) were observed for early (0.5, 1 and 3 h) and late (6, 12 and 24 h) cold treatment (4°C). AtNF-YB2 expression was downregulated during early cold stress response while upregulated at the later stages. Similar switching behaviour was displayed by AtNF-YB4 and AtNF-YB8, revealing these genes to play a putative role in late stages of plant adaptation to cold (Figure 6).

For drought, osmotic and oxidative stress responses, early and late periods of study were analysed. Early drought events resulted in the upregulation of subunits NF-YB2, B6 and B9, while later events brought about induction of B4 and B8. Similarly, osmotic stress occurrence in early phases induced TFs NF-YB2, B3, B5, B6, B9 and B10, while only B2 and B6 remained upregulated in the later time frame of sampling, while the others got downregulated. Oxidative damages in the early stages upregulated subunits NF-YB1, B3 and B5, while B1 consistently stayed upregulated in the later stages. The dynamic nature of expression switching is inconclusive as to its mechanism of action yet brings about an interesting frame of study for role mining in various stress phases to generate genome edited crops (Figure 6). Heat stress experiments showed AtNF-YB2/B4/B6/B8 and B10, showed upregulation as compared to the other subunits with AtNF-YB2 showing highest upregulation. Wounding experiments were conducted with leaves punctured with pins and then harvested at an early/late stage of 1–3 h post injury. NF-YB members (AtNF-YB5/B6/B9/B10) were upregulated while the other subunits showed medium levels of downregulation in the early stages, while AtNF-YB4/B8/B9/B10 showed induction in expression in the later harvested samples. Role-mining the regulation of TFs under abiotic stresses are imperative to understanding their role in conferring abiotic stress tolerance in plants.

Plants have endogenous small signal molecules called phytohormones that have an essential role in plant growth, development, and defence responses. Exogenous application of such phytohormones have shown to ameliorate damage caused by stress. MeJA is an essential phytohormone known to heighten plant defence response against necrotrophic pathogens. MeJA (10uM) was used to treat *Arabidopsis* (col-0) seedlings for 0.5, 1, and 3 h and expression of NF-YBs were examined. Exogenous application of MeJA heightened the transcript levels of AtNF-YB1/B4/ B9 across all three timepoints (Figure 6). Abscisic acid (ABA), similarly, is an important phytohormone that modulates growth to coordinate plant adaptation to various stresses. Treatment to *Arabidopsis* (col-0) seedlings with ABA, resulted in all the 10 genes to be modestly upregulated in different time points, inferring that the family is a participant to ABA modulation. Salicylic acid (SA) is already an important component of heat response of plants. SA plays an important role in reduction of heat stress induced cell membrane disruption, by enhancing antioxidant enzymes within plants and exogenous application has shown to alleviate plant response to stress (Shah Jahan, 2019). Many members of NF-YB were down regulated post SA treatment, indicating this family to be putatively negatively regulating SA response. Fold change values of all experimental conditions can be found in the Supplementary File 1. These selected datasets revealed the dynamic regulation of all NF-YBs in response to phytohormones and these preliminary results are essential for broad level understanding of the intricate role of phytohormones in plant defence responses and transcriptional programming (Figure 6).

**Figure 7:**
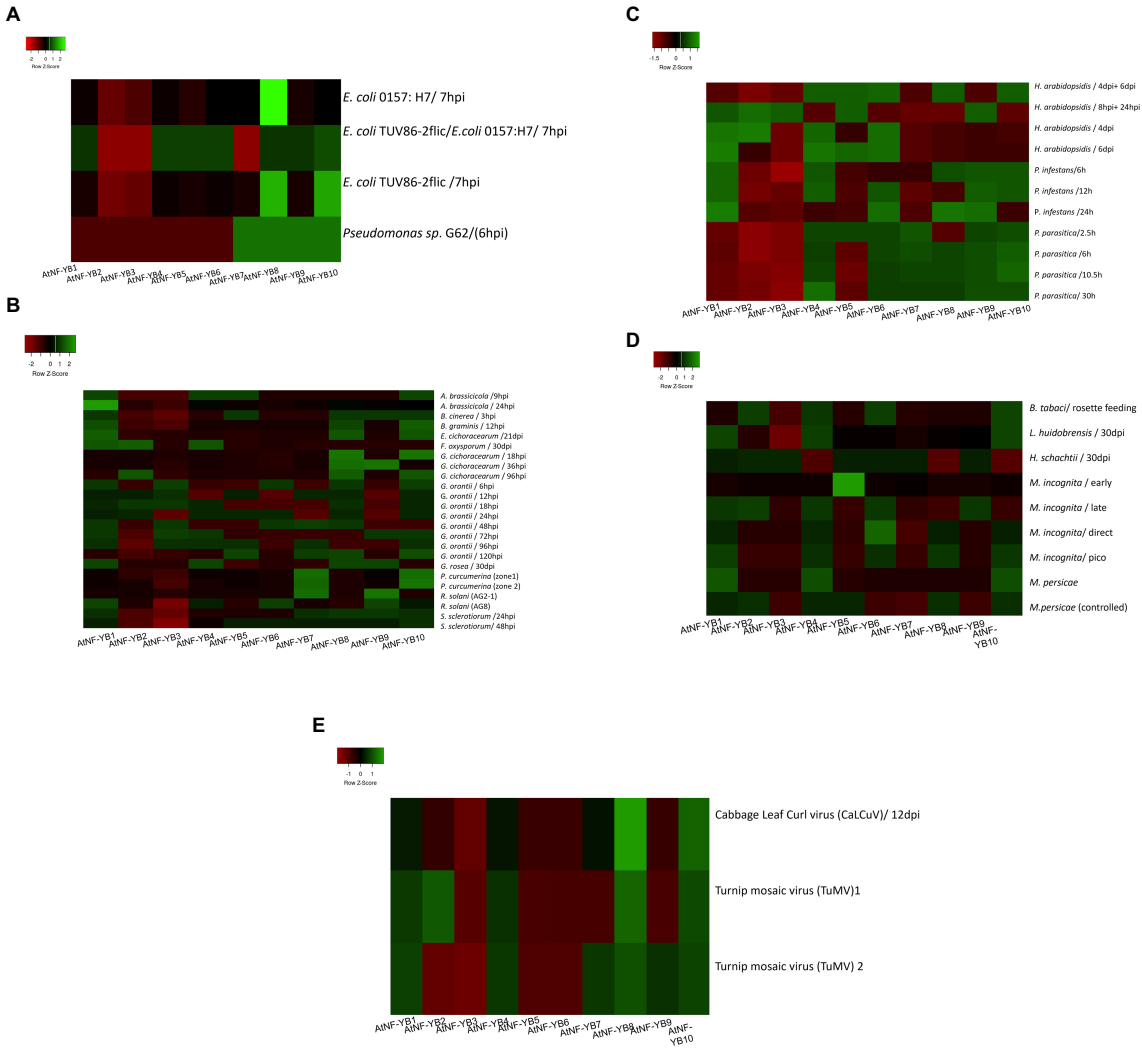
**(A)** Heat map showing the expression profiles of 10 AtNF-YB genes under different bacterial stress conditions. The colour scale is representative of the log2- transformed relative gene expression that is observed under control conditions. Red to green colours denote low to high relative expression levels, respectively. **(B)** Heat map showing the expression profiles of 10 AtNF-YB genes under different fungal stress conditions. The colour scale is representative of the log2- transformed relative gene expression that is observed under control conditions. Red to green colours denote low to high relative expression levels. **(C)** Heat map showing the expression profile of 10 AtNF-YB genes under different oomycete infections. The colour scale is representative of the log2- transformed relative gene expression that is observed under control conditions. Red to green colours denote low to high relative expression levels. **(D)** Heat map showing the expression profile of 10 AtNF-YB genes under different pest stressors. The colour scale is representative of the log2- transformed relative gene expression that is observed under control conditions. Red to green colours denote low to high relative expression levels. **(E)** Heat map showing the expression profile of 10 AtNF-YB genes under different plant virus infections. The colour scale is representative of the log2- transformed relative gene expression that is observed under control conditions. Red to green colours denote low to high relative expression levels.

Within the ecosystem, biotic stress is caused by many organisms such as bacteria, fungi, viruses, nematodes, and insects. These affect plants directly, depleting them of nutrients and many time causing death of plants. To understand how these stresses affect the plants, an understanding of the regulatory mechanisms and genes partaking in defence responses is crucial. Basal expression experiments with *Pseudomonas syringae* sp. G62 indicated severe downregulation of AtNF-YB1-B6, while AtNF-YB7/B10 showed decent overexpression (Figure 7). For necrotrophic fungal pathogen *Alternaria brassicicola*, most of the AtNF-YB genes were found to be downregulated, with only AtNF-YB1, B4, B5 and B10 showing moderate upregulation. To analyse the relationship between the fruit devouring fungus *Botrytis cinerea*, and the AtNF-YB TFs, it was observed that AtNF-YB3 showed severe downregulation, followed by AtNF-YB2/YB4/B6/B7. *Blumeria graminis* infection downregulated almost all genes, severely impacting NF-YB3, only upregulating AtNF-YB1/B8/B10. A common insect pest, silverleaf whitefly (*Bemisia tabaci* type B) infestation on *Arabidopsis* plants, caused significant downregulation of AtNF-YB3, while upregulating AtNF-YB10/B6/B4/B2. *Phytophthora infestans*, an oomycete, on infection upregulated AtNF-YB1/B4/B8/B9/B10. Viruses are obligate parasites, which are silent killers of plants and extremely difficult to handle. To assess the effect of *Turnip Mosaic Virus* (TuMV), which is an agriculturally important single stranded RNA virus, plants infected by viral transcripts were analysed for different zones. NF-YB8, exhibited strong upregulation (2.09-fold increase) followed by AtNF-YB10, NF-YB1 and NF-YB4. Similarly for the plants infected with the DNA virus *Cabbage Leaf Curl Virus* (CaLCuV), AtNF-YB8 was strongly induced amongst the entire family, indicating its role in transcriptional regulation of viral stress response (Figure 7E). All the expression data for the stress conditions from the datasets are represented in the colour coded heatmap with broadly labelled stress identity and individual fold change values are represented in Supplementary File 2. These findings indicate that the NF-YB family of TFs play very broad role in biotic and abiotic stresses; however, further experimental evidence is required to validate NF-YBs functional role in stress tolerance (Figures 7A–E).

### 3.6. Gene expression analysis revealed differential expression patterns of AtNF-YB under stress conditions

**Figure 8:**
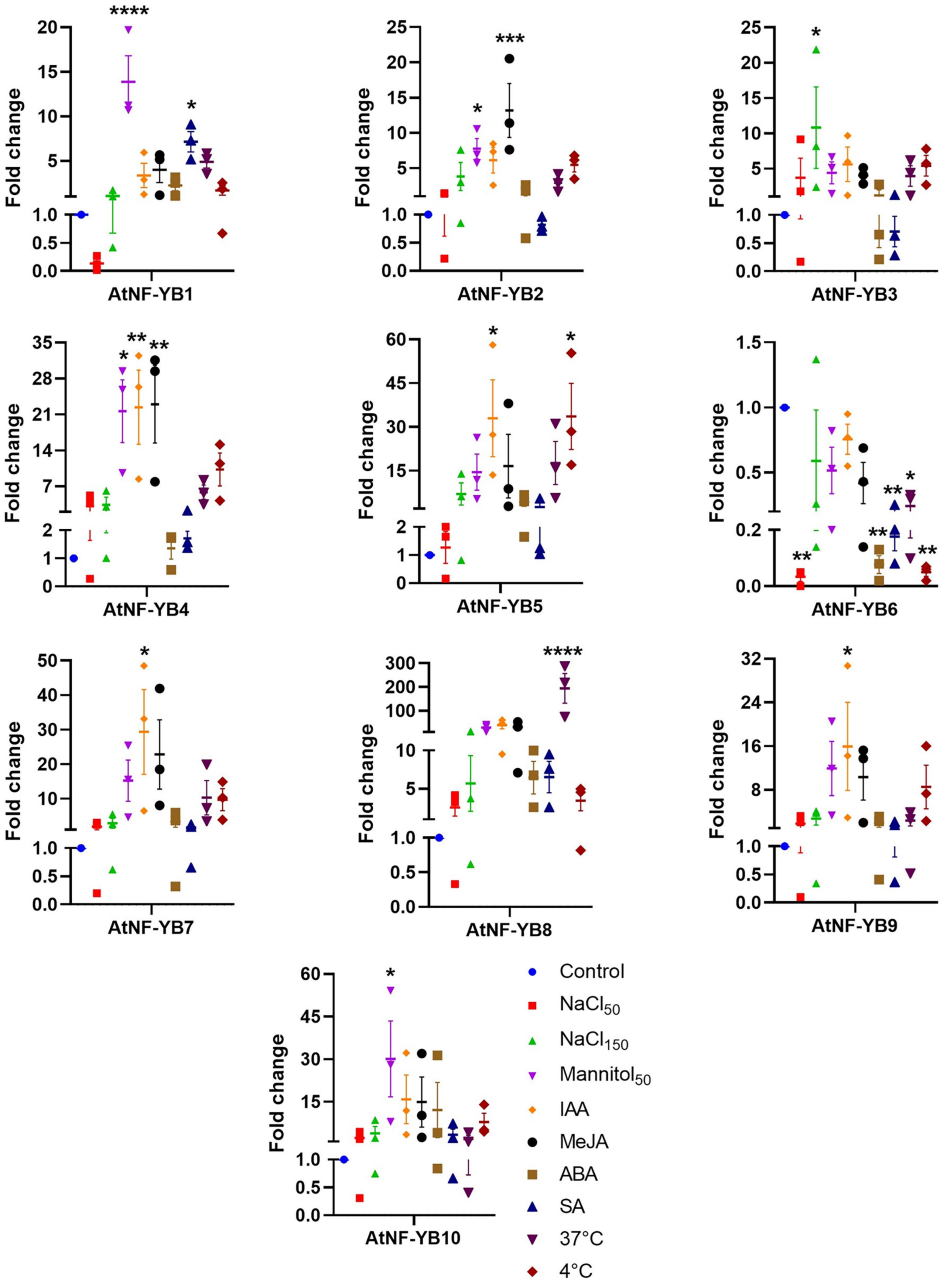
Expression analysis using qRT-PCR of all 10 AtNF-YBs under selected stressors. *Arabidopsis thaliana* (Col-0) wild type was used for control and treatment experimental sets. Experiments were performed in triplicates and GraphPad Prism 9.4.1 was used for the analyses.

To validate the microarray data of AtNF-YB TFs under various conditions, *Arabidopsis* (col-0) seedlings were subjected to abiotic stress conditions and expression levels were compared to untreated control seedlings using qRT-PCR (Figure 8). We found that AtNF-YB1 expression was significantly increased under 50 mM mannitol and 10 μM SA, with an approximate 14- and 7-fold increase, respectively. Similarly, AtNF-YB2 expression induced by 8-fold by 50 mM mannitol and 13-fold by 10 μM MeJA inferring that these subunits possibly participate in hormonal responses. Different subunits showed significant variations in transcript levels (Figure 8). Interestingly, amongst the different factors analysed, mannitol and indole acetic acid (IAA) led to the most significant changes. It is worth mentioning that various treatments led to a significant reduction in AtNF-YB6 expression, which may suggest that the transcriptional regulation of this gene may be abiotic stress independent in plants. Furthermore, as implied in Figure 8, AtNF-YB subunits were found to show differential gene expression patterns. In a nutshell, IAA and mannitol were responsible for affecting transcriptional changes in most of the subunits. It is to be noted that plants fine-tune the expression levels of AtNF-YBs to cope with different stresses at different time periods and there, the expression levels have veritable changes in time and response dependent manner.

## 4. Discussion

When under biotic or abiotic stress, plants modify cellular, biochemical, molecular, and physiological responses. Transcriptional activators and repressors, which regulate downstream stress-responsive genes that control various metabolic and defence-related activities within the plant system, have an impact on these modulation types (Tolosa and Zhang, 2020; Bhattacharjee and Hallan, 2022). Comprising of three families, namely NF-YA, NF-YB and NF-YC, these are widespread across eukaryotic families. The present study exhaustively covers the characterization of NF-YB family subunits of *A. thaliana*. An evolutionarily conserved TF family, much of its specific roles are understudied and yet to be unearthed. The study was undertaken to understand the characteristics of this family with regards to gene structure, phylogeny, expression pattern in response to various biotic and abiotic stresses and CRE enrichment in the promoters. The NCBI tool was used to investigate basic properties of all the 10 subunits. The 10 NF-YB TFs are spread across all the 5 chromosomes of *A. thaliana* with majority of them being in chromosome 2, followed by chromosome 5 and 1. Visual representation of the chromosomal locations were conducted by chromosome map tool (TAIR; Figure 1A). Locus ID, gene length and amino acid length was also derived from NCBI (Table 1). We also characterised the physicochemical properties like GRAVY, pI, instability index using ProtParam tool of ExPASY. Molecular weight of the proteins ranged from the smallest at 15.74 kDa of AtNF-YB4 to the largest at 26.1 kDa of AtNF-YB6. Through the instability index and GRAVY, we deduced that AtNF-YB1 to AtNF-YB5 were stable proteins under constitutive environments, whereas AtNF-YB6 to AtNF-YB10 were unstable proteins. All the subunits were found to be putatively nuclear localised (Table 2). Understanding and classifying phylogenetic trees are important to understand gene roles, their ancestral convergence and divergence and the evolution of their functionality and therefore, we categorised the 10 TFs in a phylogram, representing evolutionary lineages based on genetic change (Figure 1B). The MEME programme (Bailey et al., 2006) and MOTIF Search tools, provided to us the information of motif locations in the AtNF-YB protein family. Most of the motifs identified in these families were histone motif families and chromatin binding motifs, which sheds light on the role of these TFs on transcription and regulation of cellular activities, making these a very important class of proteins for appropriate cellular functioning.

Promoters hold the “key” to the transcriptional “lock” of a gene. Using the AtcisDB tool, which is a formidable tool, promoter regions predicted were isolated for all individual AtNF-YBs and the length and overall position was annotated (Table 4). A wide range of promoter elements were discovered using PLANTCARE, which dropped a lot of insight into the regulatory elements and their putative roles. The huge cache of elements primarily fell in to two categories: stress and development related. The variety of these elements across the entire family was noticed to be very diverse, inferring that these NF-YBs have an essential role in multitude of pathways in the plant (Figures 3, 4).

In this study, we impressed upon the basic expression studies of these factors and found veritable and diverse results. Almost all AtNF-YBs express constitutively at a basal level across all development pathways in the plant, ranging from seeds to senescence. TFs have important regulatory roles and are also essential participants to plant defence responses against abiotic and biotic stresses. TFs also work in tandem with exogenous phytohormones to enhances its defensive capacities. The expression analysis of AtNF-YB genes from the Genevestigator microarray datasets are indicative of the fact that these are highly variable in expression patterns. Almost all AtNF-YBs express constitutively at a basal level across stage specific pathways, ranging from germinated seeds to senescence (Figure 5). AtNF-YB1 and AtNF-YB10 were found to be strongly expressed during senescence and AtNF-YB6 in mature siliques, indicating a likely role in dry silique and pod shattering events. In the various developing stages of *A. thaliana*, AtNF-YB2 and AtNF-YB3 showed a consistent range of induction of expression and is important for regulation of flowering time by early long day photoperiods while interacting with other proteins of the AtNF-Y family of TFs (Kumimoto et al., 2008; Hwang et al., 2016). Interestingly, a progressive decline of expression was observed for AtNF-YB2 from developed flower stage, completely diminishing in the senescence stage (Figure 5). Datasets for various abiotic and biotic stresses were analysed and effectual transcriptional changes were observed, with many of the AtNF-YBs have a cluster of similar expression levels at a time, or some highly overexpressing and another severely downregulated. In the case of heat stress, it was observed that AtNF-YB2 and AtNF-YB8 showed significant upregulation, which was also confirmed by gene expression analysis (Figure 8). The presence of CREs important for stress responses like GAP-box, MYB, MYC, HSEs in the promoter regions of these two genes also affirmed their plausible roles in heat stress. Similarly, we found AtNF-YB1 promoter enriched with a host of CREs, especially those participating in stress responses like ABREs, AT-TATA, CGTCA which was highly upregulated under mannitol stress (Figure 8). AtNF-YB6 showed a trend of downregulation in datasets where exogenous application for various hormones like IAA, MeJA, SA were used, which could also be attributed to the fact that comparatively lower clusters of stress responsive CREs were seen in its promoter region (Figure 6). In case of the biotic challenges, AtNF-YB8 and AtNF-YB10 showed consistently high and broad-spectrum induction in case of different bacterial, fungal, and viral infections, followed by AtNF-YB1. AtNF-YB2 and AtNF-YB3 were substantially downregulated in *Escherichia coli* and *Pseudomonas* bacterial infections. These results indicate that AtNF-YBs have a differential role in both abiotic and biotic stress signalling and the need to analyse the basal regulatory network pertaining to these stressors and their effect in this family of TFs need to be extensively studied (Figures 7A–E).

Stress induced expression profiles of the AtNF-YB genes were determined by qRT-PCR (Figure 8). Significant differential expression patterns were seen under abiotic stress conditions, which may indicate that different members may functionally express in different stresses at different time points (Maheshwari et al., 2019). Over the years, genome-wide analysis studies have been carried out on the characterization and functional role-mining of NF-Ys in crops, e.g., *Triticum aestivum* (Stephenson et al., 2007), Barley (Panahi et al., 2019), *Citrus sinensis* and *C*. *clementina* (Pereira et al., 2018), *Solanum tuberosum* L. (Xuanyuan et al., 2022), *Fagopyrum tataricum* (Zhang et al., 2022) and *Brassica napus* L. (Xu et al., 2014), which inferred on the veritable roles of these TFs in various abiotic and biotic stress tolerance responses. While we focused on the NF-YB subunits of *A. thaliana* in this work, we found functional conservation to a certain level in our microarray and qRT-PCR data with the other published datasets. While Xu et al. (2014) showed high expression levels of BnNF-YB3, BnNF-YB7, BnNF-YB10 and BnNF-YB14 in *Brassica napus* L under salt stress, it was observed in our microarray data analysis that AtNF-YB2/3/5/9 showed significant overexpression on early stages of NaCl early-stage stress response, which change drastically at later stages where most of the genes were downregulated, inferring on the stress progression and time dependent mode of action across species. When we subjected col-0 seedlings to 50 and 150 mM NaCl stress, we observed increased transcriptional upregulation of AtNF-YB2/3/5/8/9 at 150 mM concentration, while 50 mM NaCl did not induce significant expression of majority of the subunits. It was reported that several NF-YB subunits in *Sorghum bicolor* were modulated by salt, heat, cold, osmotic and phytohormone stresses (Maheshwari et al., 2019) and noticeably, we also observed tempering of different NF-YBs at different stress responses like B9 in IAA, B8/9 in heat, B4 in mannitol, MeJA and IAA and low temperatures showing the flexibility of this group of TFs in many different classes of stress, be in abiotic or biotic. These classes of genes also display functional redundancy, although it is still not very clear as to the entire pathway of gene expression of these stress responsive TFs. The AtNF-YB family TFs function in a combinatorial manner both temporally and spatially and it would be very interesting to study how it functionally exhibits its roles as a complex bound to other proteins which are functional in development, stress, and defence responses within plants.

## 5. Conclusion

AtNF-YB family of TFs are an understudied family of proteins which are predicted to have multitude roles from morphogenesis, metabolism to defence responses in a plant. This report of curated data on *cis*-elements and expression studies on this class of TFs, and open-source data mining for expression analysis to provide insights on the AtNF-YB subunit’s roles in various pathways in plants. This work throws light on further research on NF-YB family members specifically and NF-Y family in general, for a deeper understanding of plant physiology, growth, and defence responses. Understanding this family of proteins may answer many major issues in stress biology. Preceding studies have thrown light on the capacity of AtNF-YB family of TFs in mitigating plant stress responses across plant families*. In silico* and qRT-PCR based gene expression analyses have provided crucial leads to understand the role of these factors in various environmentally significant stress responses, both pathogen dependent and independent. Further validation of these factors can provide a clear understanding of induced defence responses in the host plant system for developing commercially significant plant varieties through genome editing of these important host master manipulators.

## Data availability statement

The original contributions presented in the study are included in the article/Supplementary material, further inquiries can be directed to the corresponding author.

## Author contributions

BB and VH planned and designed the research article. BB drafted the manuscript and prepared the tables and figures. VH drafted and edited the manuscript. All authors have read and approved the final version of the manuscript.

## Conflict of interest

The authors declare that the research was conducted in the absence of any commercial or financial relationships that could be construed as a potential conflict of interest.

## Publisher’s note

All claims expressed in this article are solely those of the authors and do not necessarily represent those of their affiliated organizations, or those of the publisher, the editors and the reviewers. Any product that may be evaluated in this article, or claim that may be made by its manufacturer, is not guaranteed or endorsed by the publisher.

## Footnotes

^1^https://www.arabidopsis.org/

^2^http://www.expasy.org/tools/protparam.html

^3^http://www.genscript.com/wolf-psort.html

^4^http://cello.life.nctu.edu.tw/

^5^https://www.arabidopsis.org/jsp/ChromosomeMap/tool.jsp

^6^http://gsds.cbi.pku.edu.cn/

^7^http://www.ebi.ac.uk/interpro/search/sequence-search

^8^http://multalin.toulouse.inra.fr/multalin/

^9^http://www.megasoftware.net/

^10^https://ngphylogeny.fr

^11^http://bioinformatics.psb.ugent.be/webtools/plantcare/html/

^12^http://arabidopsis.med.ohio-state.edu/AtcisDB/

^13^https://genevestigator.com/gv/
